# Infants Do Not Reliably Track When Bilingual Speakers Switch Languages

**DOI:** 10.3390/bs15101427

**Published:** 2025-10-21

**Authors:** Christine E. Potter, Casey Lew-Williams

**Affiliations:** 1 Department of Psychology, University of Texas at El Paso, El Paso, TX 79968, USA; 2 Department of Psychology, Princeton University, Princeton, NJ 08540, USA; caseylw@princeton.edu

**Keywords:** bilingualism, infant looking time, language switching

## Abstract

It is a widely held belief that bilingual infants benefit from hearing each of their languages spoken by different people, as speakers could serve as a cue for separating the two languages. However, it is not yet known whether infants reliably attend to speaker-specific language use. In four experiments using looking time measures, we asked whether monolingual and bilingual infants in the U.S. could learn pairings between speakers and languages. Infants were first familiarized with two speakers, each using a different language. Then, after infants habituated, the two speakers switched languages, and we measured whether infants showed increased interest in hearing the speakers use a different language. Across all four studies, infants did not show reliable evidence that they detected a change in the language used by individual speakers, suggesting that speaker-language associations may not be a salient source of information for infants.

## 1. Introduction

In multilingual environments, infants must discover patterns of sounds, syllables, words, and sentences in each of their languages. It has been suggested that this process may be easier when each language can be associated with a particular person (e.g., when one parent speaks one language and the other parent speaks the second language). It has often been assumed that consistent speaker associations make it easier for bilingual infants to separate the languages in their environment, and bilingual parents sometimes receive the explicit recommendation that they should use a “one person-one language” strategy to best support their children’s bilingual language learning (Barron-Hauwaert, 2004; Ronjat, 1913). But despite this advice, there is little evidence that infants reliably attend to this association. In four experiments, we asked whether infants could learn pairings between speakers and languages and whether they would detect a change in the language used by a given speaker.

A known challenge in early language learning involves differentiating relevant from irrelevant variation, such as variability in tokens, speakers, or accents (e.g., Apfelbaum & McMurray, 2011; Best et al., 2009; Bulgarelli & Bergelson, 2022; Graf Estes & Lew-Williams, 2015). Early in development, infants are highly attuned to acoustic differences and readily distinguish between different speakers, as well as different languages (Bahrick & Pickens, 1988; Floccia et al., 2000; Jusczyk et al., 1992; Molnar et al., 2014; Mehler et al., 1988; Nazzi et al., 2000). For example, newborns recognize the difference between their own language and unfamiliar languages (Mehler et al., 1988) and can distinguish male vs. female voices (Floccia et al., 2000). Infants are also able to learn that particular faces and voices go together (Brookes et al., 2001), and like adults, tend to be more efficient in understanding speech produced by familiar voices (Nygaard & Pisoni, 1998; Parise & Csibra, 2012; Palmeri et al., 1993). In fact, infants and young children sometimes have difficulty ignoring differences between speakers and may attend to the properties of individual voices rather than information that is meaningful across speakers (Graf Estes & Lew-Williams, 2015; Houston & Jusczyk, 2000; Jerger et al., 1993; Quam et al., 2017; Potter & Saffran, 2015; Singh et al., 2004). Given that infants in the first months of life may hear the majority of their language input from just a few people (Bergelson & Aslin, 2017; Jayaraman et al., 2015), attention to speaker identity could be adaptive in that it might allow infants to prioritize information that is most likely to be relevant.

For bilingual infants, there could be added benefits of attending to speaker identity, as some speakers may reliably use one language, offering a potential cue to help infants separate the two languages in their environments. Studies using artificial languages to simulate bilingual experience have shown that both infants and adults may be more likely to discover the presence of two different streams of information if those streams are paired with separate voices (Gonzales et al., 2015; Weiss et al., 2009). Moreover, infants use the language that someone speaks to make social inferences and to determine from whom they want to learn (e.g., Buttelmann et al., 2013; Kinzler et al., 2007; Howard et al., 2014; see Kinzler, 2021). These findings suggest that infant listeners can form associations between individual speakers and the language they produce and exploit this information to learn.

Recently, Schott et al. (2023) conducted a series of experiments to determine whether infants would pay attention to the languages used by different speakers. They tested Canadian monolingual English-learning and French-English bilingual infants’ ability to track the use of English and French by a male and female speaker and found no reliable evidence that infants in any of three age groups (5-, 12-, and 18-month-olds) detected changes in speaker-specific language use, suggesting it may be challenging for infants to learn associations between speakers and languages. However, it is possible that infants might be more sensitive to speaker-language associations when they hear less familiar speech. Prior studies show that infants are better able to discriminate between highly familiar and novel, unrelated languages (Christophe & Morton, 1998; Gasparini et al., 2021; Nazzi et al., 2000). Therefore, we tested whether infants might be able to track the use of a familiar language vs. an entirely unknown language. In addition, infants’ attention to speech changes through development, particularly during the latter half of the first year (e.g., Houston & Jusczyk, 2000; Werker & Tees, 1984), and at this age, infants rapidly discover patterns and show sensitivity to the presence of different speakers (Bulgarelli & Bergelson, 2022; Graf Estes & Lew-Williams, 2015; Houston & Jusczyk, 2000; Saffran et al., 1996). Therefore, we chose to test learning by 7- to 10-month-olds.

In four experiments, we tested whether infants attended to associations between individual speakers and languages. Our goal was to evaluate whether infants would be able to detect changes in the languages used by individual speakers. Infants were always habituated to two speakers, a male and a female, each using a different language (see Table 1). During the Habituation phase, one speaker (e.g., the male) always used a familiar language, English, while the other (e.g., the female) speaker always used an unfamiliar language, Arabic. The pairings were then switched (e.g., the male speaker spoke Arabic, while the female speaker spoke English). This design was intended to simulate a one person-one language environment in a lab setting and to test infants’ responses when previously consistent associations were violated.

If infants are sensitive to speaker-language combinations, they would be expected to show increased interest in new pairings following the switch. Across studies, we tested different groups of infants to determine whether age (Experiments 1–2), additional visual and social cues (Experiment 3), or real-life bilingual experience (Experiment 4) would influence infants’ ability to track the languages used by individual people.

## 2. Experiment 1

Experiment 1 tested whether monolingual, English-learning 10-month-olds would show sensitivity to speaker-language pairings. We predicted that infants would be able to learn associations between the voices of particular speakers and the languages that they used, evidenced by increased attention to the speakers when these associations were violated.

### 2.1. Methods and Materials

#### 2.1.1. Participants

Participants in Experiment 1 were twenty 9- to 10-month-old monolingual English-learning infants (8 females, 12 males; mean age = 9.9 months, range: 9.2–10.9) living in the Mid-Atlantic region of the United States. All infants were full-term and reported to have normal hearing and to be exposed to English at least 85% of the time, with no exposure to Arabic or any related language. Eight additional infants were tested but excluded for fussiness (6), failure to habituate (1), or not contributing usable data for both types of test trials (1).

#### 2.1.2. Stimuli

All stimuli are available on OSF (https://osf.io/m6r5e/). Auditory stimuli were sentences recorded by two highly proficient bilingual speakers of English and Arabic (one male, one female) using infant-directed speech. Both speakers were originally from Saudi Arabia and currently living in the United States, and each reported learning both languages before age three. We chose Arabic as the unfamiliar language because it is not widely used in the local community of the participants, and it is typologically distinct from English with a different phonological inventory, maximizing the likelihood that infants would be able to easily distinguish and track patterns separately for the two languages (Potter & Lew-Williams, 2019).

We created a stimulus list of 38 sentences, and the two speakers both recorded all sentences. Each speaker recorded two tokens of each of 19 sentences (one in each language). One token of each sentence was produced in Modern Standard Arabic (طارت الفراشة تحت الشمس), and the second was an approximate translation in English (e.g., *The butterfly flew under the sun*), with the number of syllables matched across languages (see full stimulus list in Appendix A and on OSF) to ensure that amount of speech, speaking rate, and prosody were as similar as possible across languages. The male speaker used a consistent pitch across languages (English mean = 143 Hz, Arabic mean = 141 Hz), but the female speaker’s natural pitch was 10 Hz higher in English, so stimuli produced by the female speaker were adjusted to have a similar average pitch across languages (M = 200 Hz). Sentences were matched in intensity (65 dB) and edited such that all four tokens of a given sentence had the same duration (M = 2.8 s, range: 2.1–3.5 s) to further ensure that acoustic differences would be unlikely to explain infants’ listening behavior.

Each trial included one English sentence and the matched Arabic sentence produced by the other speaker, such that on every trial, infants heard both speakers and both languages. For example, infants on a single trial heard طارت الفراشة تحت الشمس and *The butterfly flew under the sun*. This was the structure for up to 19 trials. Sentences were presented in alternation separated by 500 ms, and we counterbalanced which speaker and language occurred first. A set of 15 sentences were used for the Habituation phase, and the four remaining sentences were used in the Test phase, and we created two different test sets to make sure that particular content did not drive any potential differences in infants’ behavior.

On Familiar Test trials, infants heard the same speaker-language combinations that they heard during Habituation. On Novel Test trials, the speaker-language combinations were reversed; infants familiarized with the male-English/female-Arabic stimuli heard Novel trials where the male speaker used Arabic and the female speaker used English and vice versa. Thus, materials that were Familiar for some infants were Novel for others, and all Test trials involved identical speakers and languages; only the pairings between speakers and languages differed (Table 1). This design allowed us to control for infants’ attention to idiosyncratic characteristics of the speakers’ voices as well as interest in familiar vs. unfamiliar languages. We chose to compare infants’ looking times on Familiar vs. Novel Test trials (as opposed to using a Switch procedure, where infants’ looking on Novel trials is compared to their behavior on the last Habituation trials, Werker et al., 1998) to reduce the possibility of experimenter bias during the Test phase (see below).

#### 2.1.3. Procedure

Infants were tested using the Visual Fixation Procedure (Werker et al., 1998; E. K. Johnson et al., 2011). Infants sat on their parents’ laps in a darkened, sound-attenuated room and viewed stimuli on a central 55” TV monitor with auditory stimuli played over a loudspeaker. Parents listened to music over headphones and were asked not to interfere. On each trial, the infant’s attention was attracted using a blinking light. Once the infant fixated on the screen, a scrolling checkerboard stimulus appeared, and speech was played until the infant looked away for 2 s or a maximum of 16 s elapsed. Stimuli were presented using custom Matlab software version 9.2 and controlled by a trained experimenter who wore noise-canceling headphones to ensure that they were unaware of the experimental condition.

The experiment included three phases: Practice, Habituation, and Test, and within each phase, the order of trials was randomized. On the single Practice trial, infants saw a colorful screensaver and heard music to familiarize them with the procedure. Then they proceeded to the Habituation phase, where they heard the two speakers each using one language exclusively. Half of infants heard the male speaker using English and the female speaker using Arabic, and the remaining infants heard the reversed pairing (Table 1). The Habituation phase continued until the infant’s looking time (calculated over a sliding window of three trials) was less than 50% of their looking time over the first three trials or 15 trials elapsed. Using an infant-controlled Habituation phase maximized the likelihood that infants would have the opportunity to learn the target associations but not become disinterested with the task. Once the Habituation phase ended, there were four Test trials (two Familiar, two Novel). Test trials were identical to Habituation trials in structure and presented in random order, and there was no signal to the infant that there was a change in phase. Similarly, there was no signal to the experimenter that the test trials had begun, which reduced the likelihood of subtle biases in coding infants’ looking.

### 2.2. Results and Discussion

All data are available on OSF. On average, infants habituated after 9.7 trials (*SD* = 3.2). To test whether infants successfully detected a change in speaker-language pairings, we asked whether their looking increased for Novel trials compared to Familiar trials. A one-tailed paired-samples t-test revealed no reliable difference between meaning looking times for the two trial types [Familiar: 4.9 s, Novel: 5.5 s, *t*(19) = −1.14, *p* = .13, Cohen’s *d* = 0.27], and only 11/20 infants showed the predicted preference for Novel trials. Contrary to our predictions, infants did not show evidence that they tracked the languages used by individual speakers.

While we had expected infants to be able to show evidence of learning the pairings between speakers and languages, this null result is consistent with other studies showing that toward the end of the second year, infants may become less sensitive to differences between individual voices as they learn to generalize patterns across speakers (Houston & Jusczyk, 2000; Potter & Lew-Williams, 2019). In contrast, younger infants may be more sensitive to indexical cues (Bulgarelli & Bergelson, 2022; Houston & Jusczyk, 2000; Singh et al., 2004). In our second experiment, we tested 7-month-old infants’ ability to track speaker-language associations.

## 3. Experiment 2

### 3.1. Methods and Materials

#### 3.1.1. Participants

Experiment 2 included twenty 7-month-old monolingual English-learning infants (5 females, 15 males; mean age = 7.3 months, range: 6.4–8.3) from the same community as the infants in Experiment 1. All infants had normal hearing and no significant exposure to any language other than English. Nineteen additional infants were tested but excluded for fussiness (5), failure to habituate (9), not contributing usable data for both types of test trials (4), or significant exposure to a non-English language (1).

#### 3.1.2. Stimuli

All stimuli and methods were identical to those used in Experiment 1.

### 3.2. Results and Discussion

In Experiment 2, infants habituated after an average of 8.6 trials (*SD* = 3.1). Like the 10-month-olds in Experiment 1, 7-month-old infants did not show significant differences in their looking times across trial types [Familiar: 4.6 s, Novel: 4.7 s; *t*(19) = −0.08, *p* = .47, Cohen’s *d* = 0.03], and only 12/20 infants showed a Novelty preference. Thus, Experiment 2 replicated our findings from Experiment 1, suggesting that 7-month-olds (like 10-month-olds) may not readily detect changes in speaker-language pairings.

One possible explanation for why infants failed to show evidence of detecting speaker-language changes could be that the stimuli did not include the full set of cues found in natural environments. The use of disembodied voices and uninformative visual stimuli could potentially impede infants’ ability to learn about speaker-specific language use. Prior work has shown that infants recognize associations between speech sounds and their corresponding facial displays (Brookes et al., 2001; Burnham & Dodd, 2004; Danielson et al., 2017), and that learning is more robust learning when infants are provided with multimodal and/or socially meaningful cues (Bahrick et al., 2004; Ferguson & Lew-Williams, 2016; Frank et al., 2009; Rabagliati et al., 2019). In addition, infants have been shown to display heightened interest in human faces (M. H. Johnson et al., 1991; Gliga et al., 2009), and adults can better separate different speech streams when faces are present (Mitchel & Weiss, 2010). In Experiment 3, we incorporated somewhat richer audio-visual stimuli to test whether social cues would facilitate infants’ learning. Ten-month-old infants first watched a video showing a brief interaction between the two speakers—modeled on a manipulation that has been shown to support infants’ pattern learning in prior research (e.g., Ferguson & Lew-Williams, 2016). Additionally, we replaced the neutral checkerboard stimulus (which infants had to fixate in order to hear speech) with an image of the two people speaking so that infants could see human faces throughout the experiment.

## 4. Experiment 3

### 4.1. Methods and Materials

#### 4.1.1. Participants

Twenty typically developing 10-month-old monolingual English-learning infants (12 females, 8 males; mean age = 9.9 months, range: 9.1–10.9) with normal hearing and no significant exposure to other non-English languages participated in Experiment 3. Participants were from the same community as in Experiments 1 and 2. Nineteen additional infants were tested but excluded for fussiness (7), failure to habituate (10), not contributing usable data for both types of test trials (1), or prematurity (1).

#### 4.1.2. Stimuli

**Figure 1:**
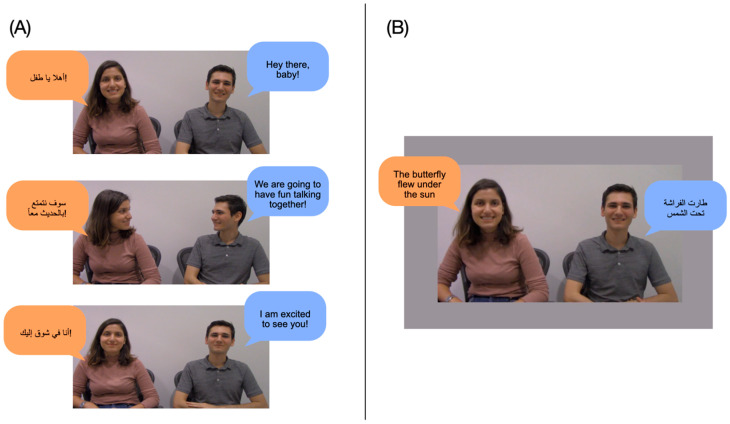
Excerpts of Familiarization video and visual stimuli used during the Habituation and Test phases, with the same content in English and Arabic. (**A**) Panel A illustrates an example conversation between the speakers in the Familiarization phase, where the male speaker is using English and the female speaker uses Arabic. Other infants saw and heard the reversed pairings. (**B**) Panel B depicts a Novel Test trial, where the speakers switched languages. On all Test trials, participants saw a still image of the two speakers, which loomed to maintain infants’ attention.

*Familiarization video.* The same two speakers who recorded the auditory stimuli were filmed sitting side-by-side to create four 16 s videos (see Figure 1A). In each video, each speaker used a single language, and the two speakers alternated sentences in child-directed speech. Because infants are more likely to show learning in a social or communicative context (Ferguson & Lew-Williams, 2016; Rabagliati et al., 2019), the speakers addressed the baby and engaged in brief conversation with one another. For example, one speaker said, “أهلا يا طفل!”, and the second speaker said, “Hey there, baby” (see videos on OSF). Across videos, we counterbalanced which speaker used which language and which person spoke first.

*Visual stimuli.* In place of the moving checkerboard used in Experiments 1 and 2, infants viewed a looming photograph of the two speakers (Figure 1B).

*Auditory stimuli*. Auditory stimuli were identical to those used in Experiments 1 and 2. Speaker-language pairings for each infant remained consistent across the Familiarization and Habituation phases.

#### 4.1.3. Procedure

Experiment 3 began with the Familiarization video. After the video, the Habituation and Test phases were identical to those in Experiments 1 and 2.

### 4.2. Results and Discussion

It took an average of 8.7 trials (*SD* = 3.3) for infants to habituate in Experiment 3. Consistent with the prior two studies, infants in Experiment 3 did not show differences in their looking times for Familiar vs. Novel trials [Familiar: 6.7 s, Novel: 7.5 s; *t*(19) = −0.72, *p* = .24, Cohen’s *d* = 0.21]. Although looking times were longer than in the first two experiments only 10/20 infants displayed a Novelty preference, suggesting that while social information (i.e., human faces) may have increased infants’ overall attention, they still did not show evidence of noticing violations in the associations between individual speakers and languages. This lack of difference is consistent with the results of Schott et al. (2023), as well as a study by Marcet et al. (2024) showing that even when infants demonstrate visual preferences for faces that have been previously paired with a particular language, they do not show learning of associations between faces and languages.

Another possible explanation for infants’ lack of sensitivity to speaker-specific language use is that in these first three experiments, we tested monolingual infants who did not have experience interacting with people who speak different languages. As monolingual infants develop expertise with the important cues in their linguistic environments, it is suggested that they show decreased sensitivity to individual speakers, particularly when listening to unfamiliar languages (Houston & Jusczyk, 2000; E. K. Johnson et al., 2011). There is evidence that bilingual infants may be more likely than monolinguals to segregate their learning by speaker (Gonzales et al., 2018), and multilingual experience can affect the linguistic distinctions that infants attend and guide their expectations about the useful cues in their environment (e.g., Comishen et al., 2019; Ferjan Ramírez et al., 2017; Petitto et al., 2012; Sebastián-Gallés et al., 2012; Singh et al., 2015, 2023). Therefore, in Experiment 4, we recruited bilingual infants to test whether regular exposure to speakers who use different languages would influence infants’ learning.

## 5. Experiment 4

### 5.1. Methods and Materials

#### 5.1.1. Participants

Twenty typically developing 10-month-old bilingual infants (9 females, 11 males; mean age = 10.1 months, range: 9.1–11.8) from the same community as participants in Experiments 1–3 participated in Experiment 4. All bilingual participants were reported to have normal hearing and to be regularly exposed (20% or more of the time) to English and at least one additional language (mean English exposure: 48%, *SD* = 17%, range: 20–80%). To be eligible, bilinguals’ non-English language could not be Arabic or any related language (e.g., Hebrew) to ensure that English was familiar and Arabic was unfamiliar to all participants. Bilingual infants’ primary non-English languages (as reported by parents) were Spanish (7), Chinese (2), German (2), Cantonese, French, Italian, Japanese, Mandarin, Polish, Portuguese, Russian, and Tamil. Twenty-one additional participants were tested but excluded for fussiness (2), failure to habituate (8), not contributing sufficient usable data (1), parental interference (2), equipment issues (5), or not meeting language eligibility criteria (3).

#### 5.1.2. Stimuli

All stimuli and procedures were identical to those used in Experiments 1 and 2.

### 5.2. Results and Discussion

**Figure 2:**
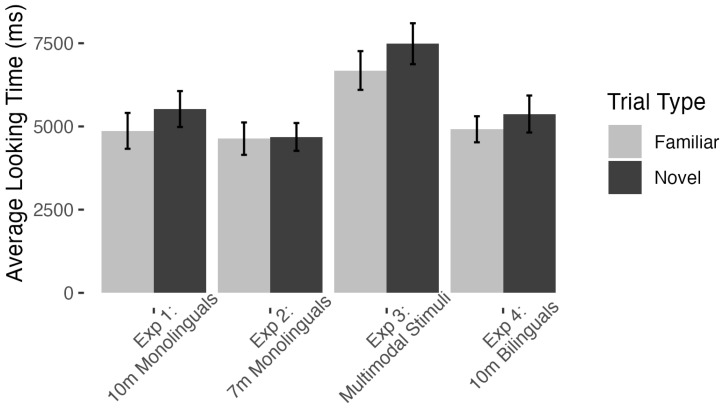
Infants’ mean looking times on Test trials across experiments. Error bars indicate standard errors of the mean.

In Experiment 4, infants completed an average of Habituation 9.1 trials (*SD* = 3.3). At Test, bilingual 10-month-old infants, like monolinguals, did not show significant differences in their looking to Familiar vs. Novel trials [Familiar: 4.9 s, Novel: 5.4 s, *t*(19) = −0.72, *p* = .24, Cohen’s *d* = 0.21, Figure 2], with 12/20 infants showing a Novelty preference. While bilingual infants have sometimes been shown to detect regularities that monolinguals do not (Antovich & Graf Estes, 2020; Kovács & Mehler, 2009), we found no evidence that bilinguals were more likely to track speaker-language associations. These results are consistent with other studies showing that bilingual experience may not boost infants’ ability to learn these associations at either younger or older ages (Birulés et al., 2024; Schott et al., 2023).

Across four separate experiments, we found the same pattern of behavior: Infants did reliably not dishabituate to switches in speaker-language pairings (see Figure 2). Even when we collapsed data across experiments, a mixed (Experiment by Trial type) ANOVA showed that there were differences in overall looking time across experiments [*F*(3, 76) = 5.43, *p* = .002, η_p_^2^ = .18], but there was no significant effect of Trial type [*F*(1, 76) = 1.61, *p* = .21] and no interaction between Experiment and Trial type [*F*(3, 76) = 0.18, *p* = .91]. Even using the most liberal test we could reasonably use, a paired-samples one-tailed t-test that included all 80 infants, there was no significant difference in infants’ looking to Familiar vs. Novel trials [(*t*(79) = −1.29, *p* = .10, Cohen’s *d* = 0.18]. This consistent lack of difference supports the view that infants do not attend closely to or readily detect changes in pairings between speakers and languages.

## 6. General Discussion

In four experiments, we examined infants’ ability to associate speakers with the languages they use. Despite many prior studies demonstrating that infants are highly sensitive to characteristics of both the languages and voices in their environment (e.g., Christophe & Morton, 1998; Mehler et al., 1988), participants in our experiments did not demonstrate the ability to learn deterministic associations between speakers and languages. Alternatively, even if they did learn these associations, there was no evidence that they expected speakers to remain consistent in their language use. While this lack of sensitivity was unexpected, these results are consistent with the view that infants do not weigh all the cues in their auditory input equally and may focus less attention on information that has not been meaningful in their prior perceptual or social experience (Lewkowicz & Ghazanfar, 2009; Potter & Lew-Williams, 2019). Thus, rather than reflecting a “failure” of learning, infants’ behavior can instead be interpreted as the development of adaptive learning strategies that allow them to manage the complexity of multi-talker, multilingual environments.

The fact that infants did not appear to notice when a speaker changed languages was somewhat surprising, given that both children and adults are known to incorporate speaker-specific expectations into their processing of speech (e.g., Borovsky & Creel, 2014; Creel, 2018; Nygaard & Pisoni, 1998) and that infants have been shown to have difficulty generalizing information across speakers in lab tasks (Graf Estes & Lew-Williams, 2015; Houston & Jusczyk, 2000). Likewise, prior research has reliably shown that infants much younger than those that we tested can reliability differentiate between languages (e.g., Hesketh et al., 1997; Mehler & Christophe, 1995; Nazzi et al., 2000), yet infants in our studies did not appear to link familiar vs. unfamiliar languages with particular speakers. Thus, while infants may be able to discriminate between languages, they do not appear to readily associate those languages with different people.

One possibility for the apparent lack of learning is that we did not measure infants’ sensitivity to any content of the speech. Studies that have documented infants’ segregation of their learning by speaker have tended to test whether infants recognize specific words or patterns that had been repeatedly produced by a particular speaker (Gonzales et al., 2015, 2018; Houston & Jusczyk, 2000). Here, although the stimuli consisted of real grammatical sentences, each trial included unique content, and the speech may not have had the same meaning for infants. That is, we were not testing their ability to identify the same speaker repeating the same speech, as in many prior studies. Infants are known to pay more attention to speech that contains learnable regularities (Gerken et al., 2011), and they may not have attended long enough to demonstrate learning of speaker-language associations at test. In addition, other studies have also shown that speaker cues may not suffice to help infants separate and learn two sets of patterns (Benitez et al., 2020; Potter & Lew-Williams, 2019), and with the complexity of multiple speakers, multiple languages, and a variety of different tokens, the task may have simply been too difficult. Future studies could test whether using simpler and/or more repetitive stimuli might allow infants to detect similarities that would enable them to learn these associations.

While this challenging aspect of the task could have contributed to our repeated null results, the fact that infants of different ages and language backgrounds showed similar behavior suggests that their input does not necessarily highlight speaker-language associations, and this kind of association may simply not be crucial to pay attention to and track. There is other evidence that as infants gain expertise with language, they learn to ignore indexical variation and focus on phonological, prosodic, or linguistic information that is more likely to carry meaning (e.g., Rost & McMurray, 2009; Thiessen & Saffran, 2007). When infants in our experiments were hearing a highly familiar language (English) and a language that was entirely novel (Arabic), they may have been, for example, more focused on the differences between the two languages than on the voices producing those languages. Moreover, infants are unlikely to have the expectation that speakers will spontaneously change languages, especially if they are raised in monolingual environments where all speakers use the same language. But even in bilingual environments, speakers do not tend to switch languages at random. Though most bilingual parents do report using both languages (Byers-Heinlein, 2013), even high estimates suggest only about 5–15% of bilingual input includes switching, and many reports are significantly lower (Bail et al., 2015; Luo et al., 2020; Smolak et al., 2020). In addition, there is significant variability in how that switching occurs, and individual speakers vary in the consistency of their language use (e.g., Kosie et al., n.d.; Luo et al., 2020), and even in homes where parents try to employ a one person-one language strategy, it is unlikely that they will be perfectly consistent in using a single language. We did not collect detailed measures about the language experience of the bilingual infants who participated in Experiment 4, such that we do not know how their language experience was divided across speakers. It could be that infants whose caregivers more closely adhere to a one person-one language approach would be more successful in this task, and future studies could try to link infants’ individual experiences with the kinds of regularities that they attend to and learn.

It is also important to acknowledge that infants’ performance in these studies does not necessarily imply that they cannot detect changes in the languages used by individual speakers in natural environments. Indeed, as with many experiments, our paradigms were lab simulations of multi-talker, multi-language environments. Infants received only brief exposure to the stimuli, while in real bilingual environments, infants would have many more opportunities to hear people use languages and learn associations. In addition, as discussed in Experiment 3, the task did not include many of the perceptual and social cues known to be useful for infants’ learning. Another key consideration is the fact that we had to exclude the data from 46% of the infants who were initially tested, and the most common reason for those exclusions was a failure to habituate (28/67 exclusions). This is a high rate of exclusion, but it is similar to the rates of other studies using similar tasks and measures (e.g., Bahrick & Pickens, 1988: 37%; Birulés et al., 2019: 29%; Molnar et al., 2014: 40%; Schott et al., 2023: 42%). While it is a clear limitation that we cannot meaningfully analyze data from these infants, the fact that many of them did not habituate to the stimuli may actually provide more support for the view that it was challenging for infants to learn the associations of interest. In future studies, it might be possible to increase infants’ exposure to the target pairings in order to highlight social information in natural conversation, or to expose infants to language use in a more meaningful context, such as labeling objects or events.

## 7. Conclusions

Ultimately, our claim is not that infants are incapable of learning associations between speakers and languages, or that learning these associations cannot be beneficial. Instead, we suggest that speaker-language pairings are not as readily learned as some other types of patterns that infants perceive in their language input. Across the majority of language environments, there are unlikely to be deterministic correlations between speakers and languages, and infants’ apparent lack of attention to this dimension of their input may reflect their preferential attention to regularities that are consistently useful for communicating and learning.

## Figures and Tables

**Table 1:** Speaker-language pairings that infants heard during the Habituation and Test phases.

	Familiar Pairings	Novel Pairings
Order A	English (male) Arabic (female)	English (female) Arabic (male)
Order B	English (female) Arabic (male)	English (male) Arabic (female)

*Note.* Infants heard only the Familiar pairings in the Habituation phase and were tested on both types of trials in the Test phase.

## Data Availability

All stimuli and data are available through the Open Science Framework: https://osf.io/m6r5e/.

## Appendix A. Stimuli

### Appendix A.1. Arabic Sentences

1. كانَ أخي مَحبوباً وناجحاً.
2. أُحبْ أستاذي، مِثالي على الدوام.
3. المستقبلُ أمامَنا جميل.
4. هَذا صديقْ خالي، وهُما يسافرانِ.
5. يبدو أنَّ الحْيَوانات تحتاجُ لمَلِك.
6. أحضرتُ طعامي معي، وأنا جاهز.
7. أربعُ تُفَّاحاتٍ تكفيكْ اليوم.
8. هَذا نَشيدْ بلادي، فما أجمَلَه!
9. كانَ البابُ كبيراً، وكانَ فتحُه صعباً.
10. أنا أسمعُ صوتاً! فَمَنْ بالباب؟!
11. نافورةُ الماءْ تدورُ وَسَطَ البِرْكة.
12. طارت الفراشة تحت الشمس.
13. بلغَ زِلزالُ البارحةِ أقصى درجاتِه.
14. واجبُكَ المدرسيُّ صحيح.
15. أجملُ الأيَّامْ عُطلةُ الأسبوع.
16. في مُنخَفَضِ الوادي تتجمَّعُ الأمطار.
17. يتجمَّدُ الماءْ عِندَ الدَّرجة.
18. هَلْ تَذكُرونَ مدرسَتَنا الابتدائيَّة؟
19. أيَّ فريقْ سيفوز يا تُرى؟

### Appendix A.2. English Sentences

1. My brother was loved and successful in school.
2. I love my teacher, and he is always perfect.
3. The future ahead is bright and beautiful.
4. This is my uncle’s friend, they are traveling together.
5. It appears that the animals need a single king.
6. I brought my food with me, and I am very ready.
7. Four red apples are enough for you today.
8. This is my home country’s anthem, how beautiful!
9. The big door was heavy and opening it was very tough.
10. I hear a loud sound, who is at the front door?
11. The water fountain was always running in the pond.
12. The butterfly flew under the sun.
13. The horrible hurricane yesterday was of very high risk.
14. Your schoolwork is well-done and accurate.
15. The most beautiful days are the weekend.
16. The rain gathered at the very bottom of the valley.
17. Water will freeze at this exact temperature.
18. Do you happen to remember our old primary school days?
19. Which team do you think will end up winning?
